# Molecular Regulation of Concomitant Lower Urinary Tract Symptoms and Erectile Dysfunction in Pelvic Ischemia

**DOI:** 10.3390/ijms232415988

**Published:** 2022-12-15

**Authors:** Tufan Tarcan, Han-Pil Choi, Kazem M. Azadzoi

**Affiliations:** 1 Department of Urology, Marmara University School of Medicine, Istanbul 34890, Turkey; 2 Department of Urology, Koç University School of Medicine, Istanbul 34010, Turkey; 3 Proteomics Research Laboratory, VA Boston Healthcare System, Boston, MA 02130, USA; 4 Departments of Urology and Pathology, VA Boston Healthcare System and Boston University School of Medicine, Boston, MA 02130, USA; 5 Building 1A, Room 317 (151), VA Boston Healthcare System, 150 South Huntington Avenue, Boston, MA 02130, USA

**Keywords:** lower urinary tract symptoms, erectile dysfunction, bladder, prostate, atherosclerosis, ischemia, oxidative stress

## Abstract

Aging correlates with greater incidence of lower urinary tract symptoms (LUTS) and erectile dysfunction (ED) in the male population where the pathophysiological link remains elusive. The incidence of LUTS and ED correlates with the prevalence of vascular risk factors, implying potential role of arterial disorders in concomitant development of the two conditions. Human studies have revealed lower bladder and prostate blood flow in patients with LUTS suggesting that the severity of LUTS and ED correlates with the severity of vascular disorders. A close link between increased prostatic vascular resistance and greater incidence of LUTS and ED has been documented. Experimental models of atherosclerosis-induced chronic pelvic ischemia (CPI) showed increased contractile reactivity of prostatic and bladder tissues, impairment of penile erectile tissue relaxation, and simultaneous development of detrusor overactivity and ED. In the bladder, short-term ischemia caused overactive contractions while prolonged ischemia provoked degenerative responses and led to underactivity. CPI compromised structural integrity of the bladder, prostatic, and penile erectile tissues. Downstream molecular mechanisms appear to involve cellular stress and survival signaling, receptor modifications, upregulation of cytokines, and impairment of the nitric oxide pathway in cavernosal tissue. These observations may suggest pelvic ischemia as an important contributing factor in LUTS-associated ED. The aim of this narrative review is to discuss the current evidence on CPI as a possible etiologic mechanism underlying LUTS-associated ED.

## 1. Introduction

Aging is a well-known risk factor for lower urinary tract symptoms (LUTS) and erectile dysfunction (ED) in men [1,2,3,4,5,6,7]. These conditions have an enormous economic impact on health care systems and lead to significant morbidity and even mortality in the elderly population [6]. Unfortunately, pathophysiological mechanisms underlying these urological conditions are poorly understood, preventing the development of effective protective and therapeutic measures. The concept of atherosclerosis-induced chronic pelvic ischemia (CPI) has been introduced to elucidate potential pathophysiological mechanisms involved in LUTS-associated ED in the aging male population. Atherosclerosis-induced CPI was first proposed to be a nominator of both LUTS and ED in 1998 supported by observations in an animal model that mimics the atherosclerotic ischemic condition in the human pelvis. The present narrative review aims to summarize clinical association of vascular disorders and LUTS-associated ED, basic research evidence suggesting a link between CPI and lower urinary tract dysfunction, and downstream molecular pathways contributing to simultaneous development of smooth muscle dysfunction and structural damage in the prostatic, bladder, and penile erectile tissues. These observations are analyzed as potential nominator LUTS-ED in the aging population.

## 2. Epidemiological Evidence of LUTS-Associated ED and Its Correlation with Cardiovascular Risk Factors

Epidemiological studies have shown a close correlation between LUTS and sexual dysfunction in both sexes suggesting potential role of a common etiology. Indeed, the link between LUTS and sexual dysfunction in the elderly population, particularly in the presence of cardiovascular (CV) risk factors, has been well documented and widely accepted [4,8,9]. For example, the incidence of ED was doubled in men with reduced urinary stream in comparison with men who did not have urination problems [4]. Likewise, an age-adjusted correlation was reported between ED and urinary incontinence [8]. Another study reported that penile erectile dysfunction was positively correlated with LUTS where concomitant hypertension increased this correlation significantly [9].

The association of CV risk factors with LUTS and ED was reported in the noninstitutionalized elderly population [10]. Another study involving 36,042 patients suggested that the prevalence of peripheral arterial occlusive disease significantly correlates with the development of LUTS [11]. Atherosclerotic occlusive disease of the pudendal and cavernosal arteries were shown to compromise penile hemodynamic and provoke ED in elderly patients [12]. Likewise, it was reported that a history of cardiovascular disease was often associated with greater incidence of urinary incontinence [13]. A recent study has shown that severity of LUTS and storage symptoms significantly increases the Framingham risk score in men, particularly with the components of total cholesterol level and age [14]. In women, LUTS-specific storage symptoms were suggested to be risk factors for predicting future CV risk [15].

Thus, the epidemiological data provide considerable evidence suggesting potential role of pelvic ischemia in simultaneous development of LUTS and ED in the elderly population. Clinical studies using multiple approaches to human pelvic blood flow measurement have provided further support to the concept of CPI in LUTS and ED. As an example, transrectal color Doppler ultrasonographic assessment of pelvic blood flow in the elderly patients with LUTS showed a significant decrease in bladder blood flow in comparison with younger controls without symptoms [16]. Improvement of LUTS with alpha-blockers was shown to be associated with a significant improvement of bladder perfusion [17]. Direct measurements of blood flow in the human bladder have demonstrated marked ischemia with bladder filling and distention and suggested a significant correlation between ischemia and decreased bladder compliance [18,19]. The latter two studies have further shown that the human bladder undergoes cycles of ischemia and reperfusion with filling and emptying. A recent study on the perfusion patterns of men with benign prostatic hyperplasia (BPH) and those who had arterial occlusive disorders demonstrated that vascular density in the group with vascular problems was lower in the prostatic transitional zone and the corpora cavernosa [20]. In patients with BPH, the international prostate symptom score (IPSS), quality-of-life score, and the international index of erectile function (IIEF) score were significantly worse than in the control group. In the latter study, men with evidence of arterial atherosclerosis had even worse symptom scores [20].

## 3. Experimental Models of Atherosclerosis-Induced Chronic Pelvis Ischemia

An animal model of atherosclerosis-induced CPI was developed to simulate human conditions of pelvic atherosclerosis and facilitate investigation of the role of CPI in genitourinary tract dysfunction [21,22,23,24,25,26,27]. Structural and functional assessment of the CPI model has revealed that moderate ischemia promotes smooth muscle contractile reactivity of the penile corpus cavernosum [21,22], the urinary bladder [23,24], the clitoral corpus cavernosum [25] and the prostatic tissue [26,27] whereas long term severe ischemia engenders significant degenerative changes in the smooth muscle cells and nerve fibers leading to contractile failure.

The development of a reliable animal model of CPI has been challenging. Many attempts have been made including unilateral ligation of the vesical arteries which did not produce chronic bladder ischemia possibly due to angiogenesis and collateral arterial outgrowth resulting in recovery of blood supply to the bladder [28]. Bilateral ligation of the vesical arteries resulted in significant bladder ischemia leading to severe functional and morphological changes resembling bladder necrosis which is rare in human [29]. We utilized balloon endothelial denudation of the iliac arteries followed by cholesterol diet to produce diffused pelvic arterial atherosclerosis and ischemia of the pelvic organ in the rabbits and rats [21,22,23,24]. This technology resulted in varying degrees of atherosclerotic occlusive disease that seems to spread from the site of endothelial denudation in iliac arteries to the smaller pelvic arteries ultimately extending to the pudendal, vesical, and cavernosal arteries. The odds of forming new arterial collaterals in our model appears to be minimal because of diffuse nature of the atherosclerotic process and spreading of the occlusive disease to the smaller arteries [23,24]. This animal model has revealed sustained ischemia in the bladder [23,24], penis [21,22], prostate [26,27], and female genitalia, providing us with the opportunity to investigate functional changes, structural modifications, and downstream molecular pathways in CPI.

## 4. Structural and Functional Consequences of CPI

CPI associated pathophysiological changes in the bladder, penis, and prostate in the animal model depend on the duration and severity of ischemia. For example, moderate degrees of chronic bladder ischemia (40 to 60% decrease in bladder blood flow) were shown to induce sensitization of bladder smooth muscle to contractile stimuli including carbachol and electrical field stimulation (EFS) and provoke overactive bladder contraction in vivo [24]. In contrast, long term severe bladder ischemia (over 60% decrease in bladder blood flow) diminished contractile responses to carbachol and EFS and impaired detrusor contractile function [24,30]. When compared to human conditions, moderate degrees of ischemia appear to induce urodynamic changes consistent with detrusor overactivity whereas prolonged severe bladder ischemia provokes extensive degenerative changes and leads to contractile failure similar to detrusor underactivity [24]. These progressive changes in the bladder were shown to be associated with upregulation of muscarinic M2 receptor in moderate and severe ischemia and downregulation of M3 along with upregulation of M1 after prolonged severe ischemia. Loss of neural structural integrity and marked neurodegeneration were found in moderate and severe ischemia, respectively. These observations may suggest CPI as a mediating variable in progression of overactive bladder to a state of contractile failure similar to underactive bladder [30]. Meanwhile, atherosclerosis-induced moderate CPI in the animal model was associated with impairment of penile erectile responses incited by electrical stimulation of the cavernosal branch of the pelvic nerve compared to controls [20,21].

**Figure 1:**
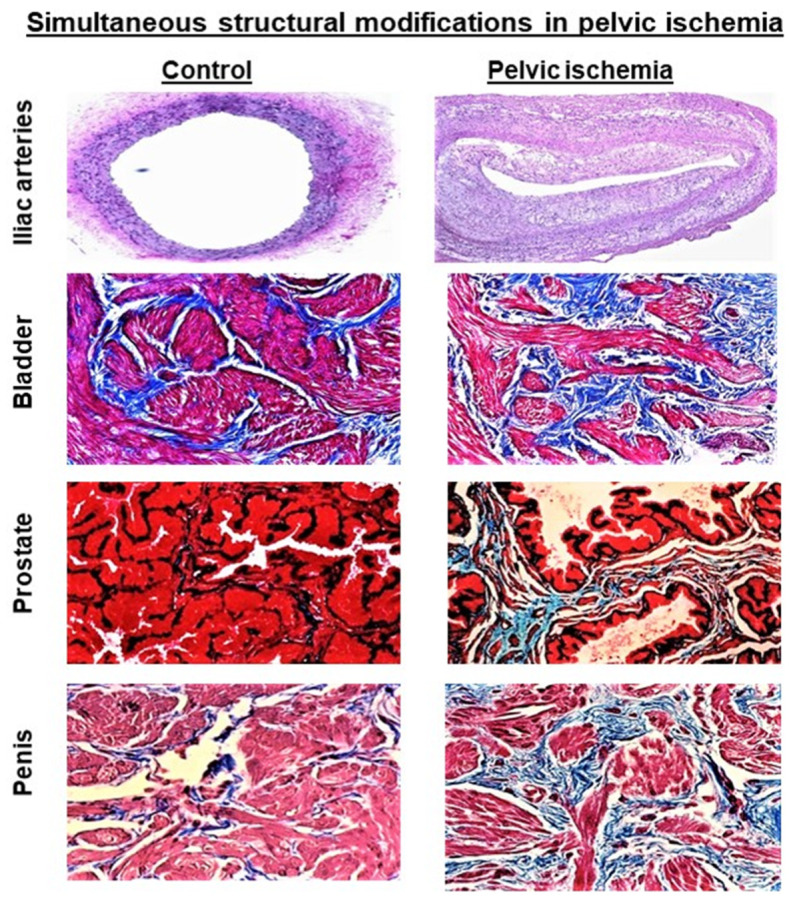
This figure shows that pelvic ischemia resulting from arterial atherosclerosis provokes simultaneous histological changes in the bladder, prostate, and penile erectile tissues. Iliac artery samples from animals with pelvic ischemia showed marked thickening of arterial intima and media layers and diminished arterial luminal size, suggesting atherosclerotic occlusive disease. Histologic assessment of tissue samples from the control bladders showed normal proportion of smooth muscle (red stain) and connective tissue (blue stain) with no indication of excessive collagen accumulation or fibrosis. Tissue samples from the ischemic bladder revealed dense bundles of collagen, suggesting diffuse fibrosis, loss of smooth muscle cells, and potential replacement of smooth muscle by connective tissue. Assessment of prostatic tissues showed swelling and thickening of the stroma, diminished smooth muscle density, accumulation of collagen, distorted glands with compressed epithelium lining, and the formation of large intraluminal spaces in the ischemic prostate tissues in comparison with control prostatic tissues. Consistent histological changes were evident in penile erectile tissue from animals with pelvic ischemia in comparison with controls. The erectile tissue smooth muscle content (stained red) was dramatically reduced, and connective tissue (stained blue) increased in the ischemic erectile tissue, suggesting loss of smooth muscle and diffuse fibrosis. The histology figures are shown at 40× magnification. These figures are taken from our previous publications [23,26,31].

Penile erectile failure in CPI was associated with upregulation of cytokines, loss of neural structural integrity, and impairment of nitric oxide (NO˙)-cyclic guanosine 3’, 5’-monophosphate (cGMP) signaling pathway due to lack of neuronal and endothelial nitric oxide synthase in the ischemic erectile tissue [20,21]. Likewise, functional assessment of ischemic tissue samples in the organ bath revealed increased contractile responses to EFS in the bladder and prostate samples and impairment of penile erectile tissue relaxation in comparison to control samples [15,16,17,18,20,21]. Increased prostatic tissue contraction was associated with impairment of cGMP synthesis [25,26]. Treatment of ischemic prostatic tissues in the organ bath with the nitric oxide (NO) precursor L-arginine, alpha1-adrenoceptor blocker doxazosin, and the phosphodiesterase-5 inhibitor sildenafil citrate diminished contractile reactivity of the ischemic prostatic tissues to the control levels [26]. Sildenafil synergized the efficacy of doxazosin in diminishing contractile reactivity of the ischemic prostatic tissues [26]. These observations may suggest the role of ischemic prostate in resistance to urinary outflow independent of prostate size. Functional changes in the ischemic bladder, prostate and penile erectile tissues were associated with marked structural modifications. Histopathological examination of the iliac arteries, bladder, prostate, and penile erectile tissues revealed marked arterial occlusive disease and consistent loss of smooth muscle cells and diffused fibrosis in tissues from the CPI model versus control tissues (Figure 1) [20,21,22,23,24,25,26]. Concurrent functional and structural changes in the ischemic bladder, prostate and penile erectile tissue may suggest CPI as a contributing factor in simultaneous development of LUTS and ED.

## 5. Molecular Regulation of Bladder Dysfunction in Pelvic Ischemia

Basic research with experimental models suggests that atherosclerosis-induced CPI provokes simultaneous structural and functional changes in the bladder [23,24], penile erectile tissue [21,22], clitoral cavernosal tissues, and prostate [26,27]. The adverse impact of CPI on the end organs depends on the severity and duration of ischemia. Moderate ischemia was shown to sensitize the bladder smooth muscle cells to contractile stimuli via multifactorial mechanisms involving cellular stress, upregulation of cellular stress sensors, upregulation of constrictor eicosanoids, and differential expression of muscarinic receptors [23,30]. These changes in moderate CPI increased the contractile reactivity of bladder tissue and led to cystometric changes consistent with detrusor overactivity [23,30,32,33,34]. However, severe or prolonged CPI compromised metabolic homeostasis of the bladder wall by reducing oxygen and nutrients levels, disturbing the bladder energy sensing system, and impairing metabolic waste clearance from the bladder. This seemed to provoke metabolic changes, enzymatic reactions, and molecular responses in the contractile components of the bladder wall, ultimately leading to loss of smooth muscle cells and the development of diffused fibrosis that appeared to be mediated by upregulation of transforming growth factor beta-1 (TGF-beta1) [23,24,30]. Structural modifications in prolonged severe CPI were associated with diminished contractile reactivity of the bladder tissue, cystometric changes simulating detrusor underactivity, and significant decrease in bladder compliance [23,24,30].

Urothelium appeared to be more reactive to ischemia in comparison with other layers of the bladder wall [23]. It was shown that CPI increases the bladder 5-lipoxygenase, cyclooxygenase-1 (COX-1), and COX-2 protein expression and alters leukotriene (LTs) and prostaglandin (PGs) production in the bladder [35]. Treatment with COX and lipoxygenase inhibitors resulted in differential changes in the ischemic bladder tissues in comparison with tissues from control bladders. Contractile changes in the ischemic bladder were associated with widespread structural damage of the urothelial layer [35]. PGs were shown to play a regulating role in contractile activity of smooth muscle cells in the healthy bladder [35]. However, in the ischemic bladder, leukotrienes appeared to modulate bladder tone and play a contributing role in increased smooth muscle contraction and the ensuing detrusor overactivity [35]. The difference between contractile reactivity of ischemic and control tissues in response to urothelial removal may suggest impairment of urothelial-mediated smooth muscle tone by ischemia [35,36]. The ischemic and control bladder tissues exhibited inconsistent responses to treatment with COX and 5-LO inhibitors. Intriguingly, chronic ischemia, urothelial removal, and indomethacin treatment produced similar changes in the contractile reactivity of bladder smooth muscle cells [35]. This observation may suggest alteration of bladder arachidonate products in favor of LTs production and regulating role of LTs in increased contractile reactivity of smooth muscle cells in the ischemic bladder.

In addition, exposure to ischemia provoked spontaneous detrusor contractions and led to repeating cycles of ischemia-reperfusion in the bladder [29,37]. Studies in metabolic cages suggested a significant increase in micturition frequency and decreased voided volume in bladder ischemia [29,37]. Conscious cystometrograms showed changes consistent with observations in the metabolic cage suggesting significant increase in micturition frequency, diminished voided volume, and reduced bladder capacity [29]. Voiding behavior and cystometric changes in bladder ischemia were associated with molecular changes characterized by significantly lower DNA binding activity of nuclear factor-erythroid factor 2-related factor 2 (Nrf2), significant increase in cellular expression of heat shock protein 70 (Hsp70) and upregulation of the mitochondrial stress proteins, namely glucose-regulated protein 75 (GRP75) [38,39,40]. Meanwhile, significant decrease in mitochondrial oxygen consumption, and upregulation of phosphatidylinositol-3-kinase (PI3K) and protein kinase B (Akt) expression were evident in the ischemic bladder [38,39,40]. A close link between overactive bladder contractions, structural damage, activation of cellular stress responses, Nrf2 functional deficit, diminished mitochondrial respiration, and upregulation of the PI3K/Akt cell survival signaling pathway was evident in the dysfunctional ischemic bladder [39]. These findings suggested that ischemia provokes cellular stress and leads to activation of cell danger patterns and cell survival signaling in the bladder.

Overactive contractions and repeating cycles of ischemia and reperfusion in the ischemic bladder resulted in the formation of oxidative and nitrosative free radicals leading to upregulation of oxidative stress sensitive genes encoding superoxide dismutase and aldose reductase [37]. Accumulation of free radicals and the ensuing cellular defensive reactions compromised ultrastructure of the subcellular elements and provoked degenerative responses in the bladder. Transmission electron microscopy of overactive bladder tissues revealed mitochondrial structural modifications characterized by swollen membranes, decreased granules or total loss of granules, and sporadic loss of membrane [37]. Mitochondrial damage in the ischemic bladder was associated with loss of epithelial structural integrity, twisted smooth muscle cells, diffused vacuolization, and diminished neural density [37]. It was suggested that diffused structural damage in the epithelium, smooth muscle cells, and loss of nerve fibers in the ischemic overactive bladder may be linked to mitochondrial stress resulting from redox and accumulation of noxious free radicals. Upregulation of superoxide dismutase and aldose reductase reported in bladder ischemia may imply activation of intrinsic defensive mechanisms against redox that seems to fail to prevent oxidative damage and neurodegeneration under the redox conditions [37].

**Figure 2:**
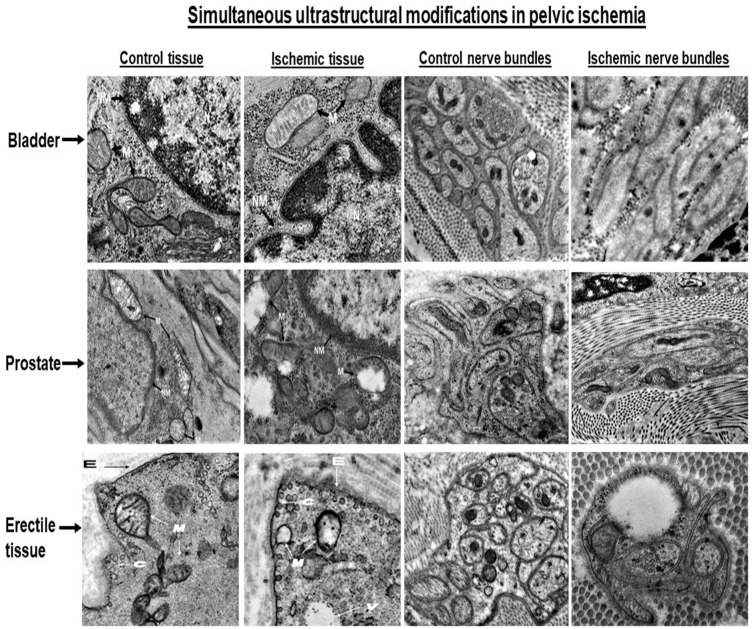
Ultrastructural assessment of bladder, prostate, and penile erectile tissues by transmission electron microscopy revealed simultaneous widespread changes in the smooth muscle and nerve bundles of the ischemic samples versus controls. Deformed muscle fascicles, increased collagen deposition, swollen mitochondria with degraded granules, partial loss of mitochondrial membrane, and sporadic vacuolization were evident in both ischemic bladder and prostatic tissues. These changes were associated with cell shrinkage, condensation and fragmentation of the nucleus, folding and partial disruption of nuclear membrane, chromatin condensation, and increased cytoplasmic and nuclear ribosomes. Loss of endothelial integrity, increased caveolae, disrupted mitochondria with degraded granules, and sporadic vacuolization were found in the ischemic erectile tissues. Normal axon terminals packed with small vesicles, and normal ensheathed Schwann cells were found in the control bladder, prostate, and penile tissue samples. Degenerating axons with collapsed axonal and Schwann cell profile surrounded by dense connective tissue, sporadic vacuoles, and splitting of myelin sheath around both axon and Schwann cell were evident in the ischemic bladder, prostate and penile erectile tissues. M = mitochondria, N = nucleus, NM = nuclear membrane, C = caveolae, E = endothelium, V = vacuole. The bladder and prostate figures are reduced from 18,500x. The penile figures are reduced from 13,000x. These figures are taken from our previous publications [37,38,39].

Molecular responses to metabolic stress and redox conditions in bladder ischemia included upregulation of gene and protein expression of hypoxia inducible factor-1alpha (HIF-1alpha), transforming growth factor-beta-1 (TGF-beta1), and nerve growth factor B (NGF) [38]. While vascular endothelial growth factor (VEGF) gene expression also increased, its protein levels remained unchanged under the ischemic conditions. These molecular responses were associated with marked ultrastructural changes in bladder microvasculature and nerve fibers [38]. Transmission electron microscopy of ischemic bladder samples showed remarkable changes in microvasculature structure characterized by thickening of intima and media and disruption of endothelial cell junctions [38]. Vascular changes in bladder ischemia were associated with diminished nerve fiber density and loss of neural structural integrity. Ultrastructural assessment of nerve fibers revealed degenerating axons and disrupted Schwann cell profiles (Figure 2). Myelin sheath splitting around axons and Schwann cells, diffuse fibrosis, and collagen invasion of smooth muscle cells and nerve fibers were evident in ischemic bladders (Figure 2) [38].

Cumulatively, these observations suggest that interruption of pelvic blood flow by arterial occlusive disease and subsequent exposure of the bladder to ischemia results in widespread structural changes that seem to be consistent with oxidative insult. Instigation of structural modifications in early-stage moderate ischemia are associated with functional changes consistent with detrusor overactivity. Degenerative changes after prolonged severe ischemia appear to deteriorate detrusor contractile activity and lead to a condition simulating detrusor underactivity. Structural modifications in bladder ischemia appears to compromise the fibroelastic properties of the bladder wall and lead to non-compliance and decreased capacity [38]. While the link between upregulation of HIF-1 alpha, TGF-beta, NGF, and VEGF and cellular and subcellular responses in bladder ischemia has been well established [38], the direct role of these growth factors in the activation of downstream mechanistic pathways mediating mitochondrial injury, fibrosis, microvasculature damage, and neurodegeneration warrants further investigation.

**Figure 3:**
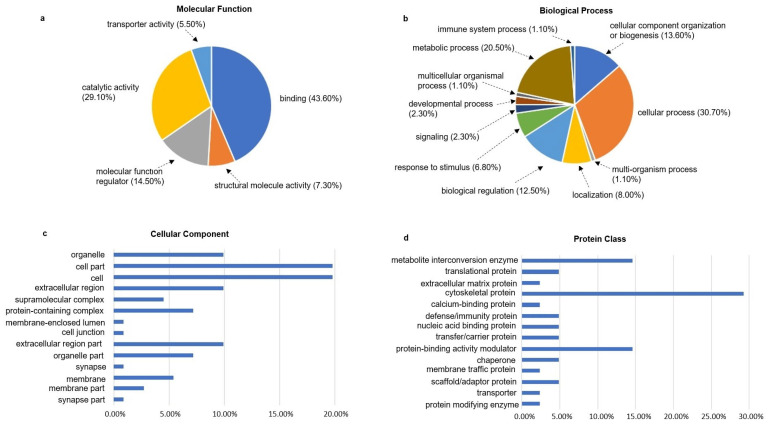
This figure summarizes gene ontology analysis of the ncAA-containing proteins in bladder ischemia. The ncAA-containing proteins (R2 > 0.5, ratio > 2-fold, *p* < 0.05) in the ischemic bladder tissues were categorized using the PANTHER database. Changes in molecular function, biological process, cellular component, and protein class are presented in sections (**a**), (**b**), (**c**), and (**d**), respectively. These figures are taken from our previous publication [41].

Proteomic analysis using Liquid Chromatography Tandem Mass Spectrometry (LC-MS/MS) revealed extensive changes in both protein expression and protein structure in the ischemic bladder tissues [39,40,41,42]. LC-MS/MS detected a total of 4277 proteins in ischemic and 4602 in control bladder tissues [39]. Analysis of the ischemic bladder tissues revealed that 359 and 66 proteins were differentially expressed with a greater than twofold and fivefold change, respectively. Ischemia provoked post-translational modifications, dominantly in contractile proteins and stress response proteins [40,41]. Gene ontology database and pathway and network analysis suggested a close link between differentially expressed proteins and molecular signaling pathways involved in the development of proteolysis, degenerative processes, ubiquitination, Nrf2 provoked oxidative stress responses, cell death, glucose metabolism and cytoskeleton remodeling [40,41]. Western blot validated changes in the expression of 4 representative proteins, including Nedd4l, Mpo, Ca3 and Fkbp5 [39]. Ischemia also engendered non-coded amino acids (ncAAs) in the bladder [41]. It is thought that formation of ncAAs may involve post-translational protein modifications and amino acid substitution mechanisms [41]. However, the precise nature of ncAAs and the specific pathways leading to ncAAs remain to be elucidated. Altered proteomic profile and post-translational modification of proteins in the ischemic bladder correlated with downstream pathways of smooth muscle dysfunction, ultrastructural modifications, fibrosis, and neurodegeneration (Figure 3) [40,41]. A summary of changes in molecular functions, biological processes, cellular components, and protein classes detected by proteomic analysis is presented in Figure 3 below. Further insight into LC-MS/MS proteomic analysis of modified proteomic profiles in bladder ischemia may help unraveling new molecular mechanisms underlying bladder dysfunction, structural modifications, and lower urinary tract symptoms in pelvic arterial insufficiency [40,41]. Additional detailed information pertaining to bladder proteome may open a new venue for future research pertaining to the verification and clinical translation of novel diagnostic biomarkers in bladder dysfunction. LC-MS/MS proteomic approach has the potential of being applied to a variety of ischemic disorders for early detection and precise analysis of disease-associated ncAAs and the development of potential therapeutic strategies.

Metabolic stress associated with ischemia was shown to compromise the bladder energy homeostasis [42]. The downstream mechanism interfering with bladder homeostasis appeared to involve adenosine monophosphate-activated protein kinase (AMPK), a key component of the energy-sensing system [42]. Analysis of ischemic bladder tissues with compromised homeostasis suggested that while total AMPK-α2 expression increases in bladder ischemia, the phosphorylated and thus activated form of AMPK-α2 is significantly downregulated [42]. Further assessment of ischemic bladder tissues using LC-MS/MS analysis implied impairment of AMPK-α2 functional domains including the phosphorylation sites [42]. The data revealed that AMPK-α2 undergoes post-translational modifications under the ischemic conditions leading to accumulation of catalytically inactive form of AMPK-α2 in bladder ischemia [42]. Treatment of bladder ischemia animals with the AMPK activator 5-aminoimidazole-4-carboxamide-1-beta-D ribofuranoside (AICAR) diminished the force of overactive detrusor contractions and increased the ischemic bladder capacity but did not have a significant effect on the frequency of spontaneous bladder contractions [42]. Histological assessment of ischemic bladder tissues implied that AICAR may protect the bladder nerve fibers against ischemic damage [42]. Studies in the organ bath produced consistent data showing that AICAR diminished contractile reactivity of ischemic bladder tissues to contractile stimuli. These observations suggest that impairment of AMPK-α2 and subsequent disruption of homeostasis in bladder ischemia may contribute to increased smooth muscle cell contractions and loss of nerve fibers in bladder ischemia. These observations cumulatively suggest that AMPK activators may have therapeutic potential against detrusor overactivity and neurodegeneration in the ischemic bladder [42].

Cell culture experiments were carried out to define responses of human bladder smooth muscle cells to hypoxic conditions and distinguish between changes induced by hypoxia versus alterations caused by hypoxia and reoxygenation. Cultured human bladder smooth muscle cells exhibited high sensitivity to hypoxia and oxidative stress conditions showing a variety of modifications at the cellular and subcellular levels [43]. Both hypoxia and oxidative stress instigated lipid peroxidation in cultured cells. However, protein oxidation was evident in oxidative stress conditions only and not in cells undergoing hypoxia [43]. Cellular antioxidant capacity was significantly impaired after cells were exposed to oxidative stress while exposure of cells to hypoxia had little impact on the cellular antioxidant capacity. Accumulation of oxidative products was found in cells exposed to oxidative stress only, whereas nitrosative products were present in both hypoxia and oxidatively stressed cells [43]. Exposure of cultured human bladder smooth muscle cells to hypoxia and oxidative stress for forty-eight hours had no significant effect on cell senescence. Transmission electron microscopy of cultured cells revealed thickened deformed cell membrane, enlarged swollen mitochondria, and enlarged endoplasmic reticulum (ER) in cells exposed to hypoxia [43]. Exposure of cultured human bladder smooth muscle cells to oxidative stress compromised cellular structure and led to partial loss of cell membrane, increased caveolae, deformed swollen mitochondria with degraded granules, loss of ER structural integrity, and accumulation of lysosomes [43]. These observations suggest that human bladder smooth muscle cells are highly sensitive to hypoxia and redox conditions. Cellular responses to hypoxia are consistent with hypoxic stress and activation of survival signaling to promote cell survival. Changes in oxidative stress imply extensive free radical mediated cell damage involving cell membrane, the nucleus, and the subcellular elements. Hypoxic and oxidative damage provoked by oxidative and nitrosative free radicals may be an important contributing factor in deterioration of subcellular elements and instigation of smooth muscle degeneration [43].

## 6. Molecular Regulation of ED in Pelvic Ischemia

Molecular aspects of ED due to arterial insufficiency have primarily been investigated in animal models. Adverse effects of atherosclerosis-induced CPI on penile hemodynamics and erectile function in an animal model were reported in 1992 [21]. Subsequently, it was shown that erectile tissue ischemia resulting from arterial occlusive disease may predict abnormal veno-occlusive function and poor erection quality [22]. Organ bath experiments using penile cavernosal tissues from the same animal model revealed diminished relaxation responses to electrical field stimulation and impairment of endothelium-dependent relaxation to acetylcholine that appeared to be due to structural deterioration of the cavernosal endothelial cells [44]. Further analysis of the ischemic erectile tissues showed that electrical field stimulation-induced neurogenic relaxation, cavernosal endothelial nitric oxide synthase (eNOS) expression and activity, and neuronal nitric oxide synthase (nNOS) expression and activity were significantly diminished through mechanisms involving hypoxia, redox, and free radicals [45,46]. Treatment with indomethacin, a non-selective inhibitor of the cyclooxygenase enzymes COX1 and COX2, increased endothelium-dependent relaxation of the ischemic and control tissues and promoted neurogenic relaxation of the ischemic tissues but failed to normalize the differences in relaxation between ischemic and control erectile tissues. Treatment with indomethacin and L-arginine, the biological precursor of nitric oxide (NO), improved endothelium-dependent and neurogenic relaxations of ischemic cavernosal tissues but did not normalize relaxation responses to the levels recorded in the control tissues [44,45,46]. Relaxation responses to NO donor sodium nitroprusside and relaxation to papaverine were similar in the ischemic cavernosal tissues in comparison with control. These observations insinuated a close link between lack of endothelium-dependent relaxation, loss of endothelial cell integrity, impairment of eNOS expression and activity, and subsequent interruption of NO production by the cavernosal endothelial cells [46]. Improvement of endothelium-dependent relaxation by L-arginine denoted lack of production or availability of L-arginine and potential interruption of NO synthesis in the ischemic cavernosal tissues. Impairment of neurogenic relaxation in the ischemic cavernosal tissue was closely linked to impairment of nNOS expression, lack of nNOS activity and disruption of neuronal NO synthesis [46]. These observations introduced the notion that pathophysiologic mechanisms in arteriogenic ED involves, in addition to disturbed hemodynamics, widespread changes in smooth muscle cells, endothelial cells and nerve fibers. In addition to surgical intervention, therapeutic strategies to improve eNOS and nNOS activity, NO production and cGMP formation may be implemented to prevent or reverse arteriogenic ED in CPI [44,45,46].

Increased output of constrictor eicosanoids in the ischemic erectile tissue may also disintegrate relaxation responses by promoting smooth muscle contractions. The basal release of constrictor eicosanoids including prostaglandin F2alpha (PGF2alpha) and thromboxane A2 (TXA2) was shown to significantly increase after erectile tissue exposure to ischemia [45]. Studies in the organ bath implied that ischemia elicits little change in contractile responses to norepinephrine while causing a significant increase in electrical field stimulation (EFS)-induced contractions. Inhibition of the cyclooxygenase pathway by indomethacin decreased EFS-induced contraction of the ischemic tissues but failed to normalize the differences between the ischemic and control tissues, suggesting the presence of additional contributing factors. Treatment with indomethacin and L-arginine caused further decrease in EFS-induced contractions in the control erectile tissue while having little effect on contractions in the ischemic erectile tissues [45]. In the presence of indomethacin, tissue treatment with the nitric oxide synthase (NOS) inhibitor, N(G)-monomethyl-L-arginine (L-NMMA) caused significant increase in EFS-induced contraction in the control erectile tissues. Treatment with L-NMMA appeared to have significantly greater effects on contractile responses of the control tissues in comparison with ischemic tissues. After tissue treatment with L-NMMA, contractile responses of control cavernosal tissues was similar to those recorded in the ischemic tissues [45]. These observations suggest sensitization of ischemic erectile tissues by constrictor eicosanoids that seems to promote contractile reactivity of smooth muscle cells while abolishing relaxation responses by disruption of the NO-cGMP pathway.

Time-dependent assessment of changes in ischemic erectile tissues revealed progressive hemodynamic, structural, and functional modifications. Studies at 4 weeks after the induction of arterial atherosclerosis revealed no significant changes in iliac artery blood flow, intracavernosal blood flow, and intracavernosal oxygen tension. However, the aforementioned parameters were significantly diminished at 8 weeks and 16 weeks after the induction of arterial atherosclerosis [44,45,46]. Congruently, erectile responses to electrical stimulation of the cavernosal nerve and relaxation responses of erectile tissue were unchanged at week 4 while these responses were significantly diminished at 8 weeks and 16 weeks after the induction of atherosclerosis-induced pelvic ischemia [44,45,46]. Molecular analysis of erectile tissues showed no significant change in cavernosal nNOS and eNOS protein expression at week 4 while they were significantly decreased at weeks 8 and 16 after the induction of pelvic ischemia [46]. iNOS protein, however, was significantly upregulated over the course of arterial occlusive disease and cavernosal ischemia. Immunohistochemical staining of erectile tissues provided consistent outcomes showing no changes in cavernosal eNOS and nNOS expression at week 4 and dramatic decreases in both eNOS and nNOS at 8 and 16 weeks after the induction of cavernosal ischemia [46]. iNOS expression appeared to progressively increase at 4, 8 and 16 weeks after cavernosal ischemia. Downregulation of nNOS and eNOS and upregulation of iNOS coincided with dramatic changes in erectile tissue tone that appeared to favor contractile reactivity while deterring relaxation responses. Differential changes in eNOS and nNOS expression versus iNOS expression over the course of cavernosal ischemia may be of great pathophysiological importance and may warrant further investigation to define the precise nature of NO produced by eNOS and nNOS versus NO engendered by iNOS [46].

Structural and functional changes in the ischemic erectile tissue were associated with accumulation of free radicals and redox [47]. Biochemical analysis and immunostaining of ischemic erectile tissues showed significant increase in oxidative products, and perceptible nitrotyrosine immunoreactivity, respectively [47]. Analysis of redox-sensitive genes encoding hypoxia inducible factor-1alpha (HIF-1alpha), superoxide dismutase (SOD), and aldose reductase (AR) suggested significant upregulation in the ischemic erectile tissues. These molecular responses were associated with loss of neural structural integrity and significant upregulation of nerve growth factor (NGF) [47]. Histological assessment followed by transmission electron microscopy showed diminished neural density, collapsed axonal and Schwann cell profiles, degeneration of nerve fibers, loss of mitochondrial membrane, degradation of mitochondrial granules, increased caveolae, disruption of the endothelial layer, and sporadic vacuolization (Figure 2) [47]. These observations introduced the notion that microvascular degeneration may precede neurodegeneration in cavernosal ischemia. Neuropathy in cavernosal ischemia appears to involve a neurovascular phenomenon involving redox, free radical insult, microvascular degeneration followed by loss of neural structural integrity [47]. Mitochondrial structural damage, increased levels of HIF-1alpha, and upregulation of NGF gene expression may be early signals of free radical insult and neural degenerative responses in the ischemic erectile tissue. Upregulation of SOD, AR and NGF may be a coordinated defensive response to promote the survival of smooth muscle cell, microvasculature, nerve fibers, and surrounding structures. These defensive responses, however, seem to fail to prevent smooth muscle dysfunction and neural injury under the hypoxic and redox conditions in erectile tissue. These observations suggest oxidative stress as a central mechanism in consecutive development of microvascular injury, endothelial damage, smooth muscle dysfunction, and neurodegeneration in arteriogenic ED. Therapeutic strategies to prevent redox and protect erectile tissue microvasculature, endothelium and nerve fibers from oxidative insult may enhance the efficacy of surgical interventions and pharmacological strategies against arteriogenic ED [47].

## 7. Molecular Regulation of Prostate Dysfunction in Pelvic ischemia

The precise mechanism by which aging prostate contributes to bladder outlet obstruction (BOO) remain unknown. A discrepancy between prostate enlargement and standardized symptom scores for obstruction and LUTS suggests that BOO could result from increased prostate tension independent of prostate size. It is widely accepted that both dynamic and static mechanisms contribute to BOO. However, dynamic forces of the prostate contributing to BOO are yet to be characterized. The underlying mechanism by which aging increases prostate tension remains essentially unknown. Studies with the animal model of pelvic atherosclerosis suggest that ischemia compromises prostate smooth muscle tone and may be a contributing factor in increased prostatic tension [48,49,50]. This notion implies that increased prostate tension in ischemia could provoke bladder outflow resistance independent of prostatic enlargement. Hemodynamic assessment of the animal model of pelvic atherosclerosis showed that interruption of blood flow through the atherosclerotic iliac arteries impair prostatic blood flow and leads to chronic prostate ischemia [48,49,50]. Histological assessment of the ischemic prostatic tissue using Masson’s trichrome staining showed structural changes characterized by stromal thickening and fibrosis, cystic atrophy of the epithelium, decreased smooth muscle cell density, and diffused fibrosis in comparison with control prostatic tissues (Figure 1) [48,49]. TEM showed enlarged mitochondria with degraded cristae, impairment of mitochondrial membrane, decreased Golgi bodies, nerve fiber degeneration, and disruption of cell-to-cell junctions (Figure 2) [50].

Exposure of prostatic smooth muscle cells to oxidative stress in cell oxycycler resulted in structural modifications similar to those seen in the ischemic prostate tissues [50]. Cell culture studies revealed that human prostatic cells exhibit differential responses to hypoxia and oxidative stress along with widespread DNA damage. Observation in the animal model and cell culture oxycycler cumulatively suggest that structural changes due to ischemia and oxidative stress may compromise the fibroelastic properties of the prostate and lead to impairment of prostatic compliance and increased prostate tension in aging men [50]. Molecular analysis suggested a close link between structural modifications of the ischemic prostate and downregulation of vascular endothelial growth factor (VEGF). Glandular atrophy in prostate ischemia was also associated with decreased VEGF expression. Ischemia appeared to sensitize prostatic tissue to contractile stimuli while impairing smooth muscle relaxation resulting in increased prostatic tension [48,49]. Functional assessment of ischemic prostatic tissues in the organ bath showed that ischemia impairs EFS-induced relaxation of the prostatic tissue while causing a significant increase in contractile responses [49]. This is consistent with changes in the ischemic penile erectile tissue described earlier. Treatment with indomethacin and L-arginine had no significant effect on the relaxation responses of ischemic prostatic tissues. The nitric oxide (NO) donor sodium nitroprusside improved relaxation responses of ischemic prostatic tissues to the control levels [49]. These observations in the organ bath suggested that lack of NO production in the ischemic prostate and subsequent impairment of the NO/cGMP pathway may play a central role in decreased relaxation in response to EFS. Cumulatively, these observations suggested that ischemia compromises the structural integrity of prostatic cells, promotes prostatic smooth muscle contractile reactivity, impairs relaxation, and leads to marked changes in the mechanical properties of the prostate. Lack of NO production and subsequent impairment of the NO/cGMP pathway may play a central role in decreased relaxation and tension development in the ischemic prostate.

In another study, cell culture experiments were carried out using a computerized oxycycler system to determine explicit responses of human prostate smooth muscle cells (SMCs), epithelial cells (ECs), and stromal cells (SCs) to hypoxia and oxidative stress conditions [31,50]. Fluorometric analysis and enzyme immunoassay were used to analyze the markers of oxidative stress in cultured prostatic cells. These studies revealed differential responses of human SMC, EC and SC to hypoxic and oxidative stress conditions [50]. For example, protein oxidation, characterized by increased advanced oxidation protein products (AOPP) levels, was present in EC hypoxia but was absent from hypoxic SMC and SC [50]. In contrast, oxidative stress, defined as repeating cycles of hypoxia and reoxygenation, led to simultaneous protein oxidation in SMC, EC and SC [50]. Lipid peroxidation, characterized by malondialdehyde (MDA) upregulation, was present in SMC exposed to hypoxia but was absent from EC and SC under the hypoxic conditions [50]. Oxidative stress produced lipid peroxidation in SMC, EC and SC. Protein oxidation and lipid peroxidation were associated with DNA damage in all types of prostatic cells examined in this study [50]. Both hypoxia and oxidative stress upregulated 8-hydroxy-2’–deoxyguanosine (8-OHdG) in the SMC, EC and SC, suggesting global DNA damage in the prostatic cells in disturbed oxygen tension. Lipid peroxidation, protein oxidation, and DNA damage in the hypoxic and oxidatively stressed prostatic cells were associated with widespread ultrastructural modifications [50]. Transmission electron microscopy of cultured prostatic cells showed that hypoxia impairs cellular membrane structure, causes sporadic loss of outer mitochondrial membrane, induces swelling and enlargement of the endoplasmic reticulum (ER), and leads to diffused accumulation of glycogen. Oxidative stress produced similar changes in the prostatic cells but in a more dramatic fashion. SMC exhibited greater sensitivity to oxidative stress characterized by extensive deformation of cell membrane, impairment of mitochondrial membrane, degradation of mitochondrial granules, enlargement and deformation of ER structure, and accumulation of cytoplasmic lysosomes [50].

**Figure 4:**
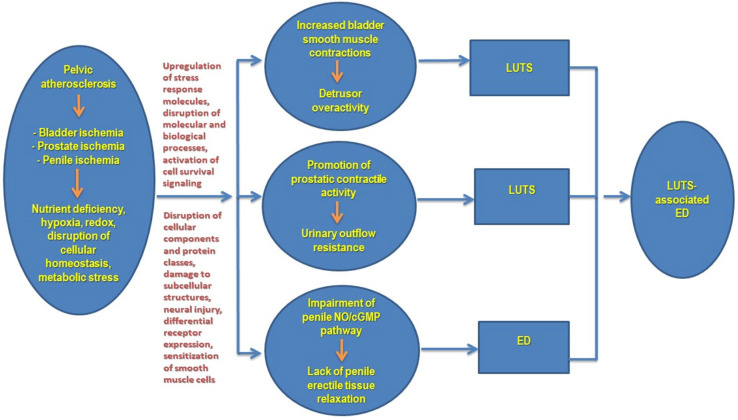
Conceptual presentation of pelvic ischemia as a unifying mechanism of LUTS-associated ED. It is believed that bladder ischemia contributes to non-obstructive non-neurogenic detrusor overactivity. Prostate ischemia promotes prostatic smooth muscle contractions and leads to urinary outflow resistance independent of prostate size. Penile ischemia disrupts the underlying mechanism of erectile tissue relaxation and impairs erectile activity. These changes, cumulatively, lead to the development of LUTS-associated ED. NO = nitric oxide, cGMP = cyclic guanosine monophosphate, LUTS = lower urinary tract symptoms, ED = erectile dysfunction.

In summary, a close link between the incidence of LUTS and ED and the prevalence of cardiovascular disease has been documented in the male patients. Atherosclerotic occlusive disease and subsequent arterial insufficiency that engenders arteriogenic ED seem to be an important contributing factor in the development of overactive bladder as well as prostatic tension. Ischemia-mediated simultaneous changes in the bladder, prostate and penis may contribute to the concomitant incidence of LUTS and ED in the aging male population. Basic research with animal and cell culture models has revealed provocation of molecular responses with adverse consequences resulting in concurring cellular and subcellular modifications in the ischemic bladder, prostate and penile erectile tissues. Functional changes provoked by ischemia seem to sensitize smooth muscle cells to contractile stimuli while impairing relaxation leading to detrusor overactivity, increased prostatic tension, and ED. Molecular mechanisms and downstream signaling pathways appear to involve cellular stress, cell survival signaling, RNA modifications, DNA damage, disproportional changes in muscarinic receptors, impairment of NO/cGMP pathway, differential protein expression, and post-translational protein modifications. These changes in conjunction with lipid peroxidation and protein oxidation activate downstream pathways involving cellular energy sensors, cellular stress response molecules, and growth factors HIF, TGF-beta, VEGF and NGF and ultimately leading to microvasculature damage, neurodegeneration, loss of smooth muscle cells and diffused fibrosis. Ultrastructural modifications include thickened deformed cellular membrane, swollen mitochondria, enlarged endoplasmic reticulum (ER), partial loss of cell membrane, increased caveolae, swollen mitochondria with degraded cristae, splintered ER, and increased lysosomes. Potential mechanisms contributing to LUTS-associated ED in pelvic ischemia is summarized in Figure 4 below.

## 8. Limitations of the Current Evidence and Future Perspectives

Current clinical and basic research evidence suggest that pelvic ischemia may be a contributing factor in simultaneous development of LUTS and ED in the male aging population. The etiological link between arterial insufficiency and ED is well established, while clinical evidence pertaining to CPI as a cause of LUTS is evolving and warrants further investigation. CPI has been well accepted as a major contributor to ED, but LUTS is traditionally attributed to bladder outlet obstruction due to prostatic hyperplasia and not atherosclerosis-induced CPI. This review along with future investigation into the role of CPI may enhance our understanding of the pathophysiology of LUTS in non-obstructed patients and could introduce new concepts in the way we counsel patients considering vascular risk factors. Gaining further knowledge into the role of CPI in genitourinary disorders could change guideline recommendations and may lead to a paradigm shift in the management of male LUTS. These may include lifestyle changes, bladder re-education, pelvic rehabilitation or medical options to improve pelvic blood flow.

In addition to age-related pelvic atherosclerotic process, future studies should also focus on amelioration of pelvic blood flow using medical therapeutic strategies including those used against BPH-associated LUTS. Current evidence suggests that, similar to alpha blockers, decreasing LUTS with PDE5 inhibitors and a beta3 adrenergic agonist mirabegron may also be related to improvement of bladder blood flow. For example, the mechanism by which the PDE5 inhibitor tadalafil improves both male LUTS and ED was shown to involve improvement of vascular elasticity and endothelial function in patients with BPH [51]. Likewise, in another study, treatment with tadalafil was considered for patients with prostatic hyperplasia that did not respond to α1-blockers [52]. Voiding symptoms were significantly improved by tadalafil, in parallel with significant improvements in vascular function, suggesting that improvement of vascular function and pelvic blood flow may play a mediating role in the improvement of LUTS by tadalafil [52]. Studies with a rat model of BOO suggested that amelioration of bladder blood flow may play a role in improvement of BOO-induced bladder dysfunction by mirabegron [53]. These observations may open new perspectives for the medical management of LUTS.

Another important field for future research would be to assess the effects of different surgical interventions for the treatment of BPH on the bladder blood flow and therapeutic strategies against LUTS by improving bladder blood flow. This may gain further significance as new surgical alternatives with potential vascular side effects are emerging in the field [54]. For example, holmium laser enucleation of the prostate (HoLEP) is shown to increase blood perfusion in the bladder mucosa and lead to improvement of storage symptoms independently [55]. Thus, preserving or improving bladder blood flow after surgical intervention for BPH may be a reliable determinant of surgical outcome.

The functional complications of pelvic surgeries such as radical cystectomy or radical prostatectomy include vasculogenic ED and sphincteric urinary incontinence. In a recent systematic review, pre-operative pelvic rehabilitation including pelvic floor exercises, vacuum therapy, low intensity extracorporeal shockwave therapy and oral PDE5 have been shown to restore erectile function. One of the mechanisms mediating pelvic rehabilitation has been suggested to involve improvement of the pelvic blood flow [56]. Further research is needed to elucidate the role of ischemia in the etiology of functional complications after pelvic surgeries and to develop preventive and therapeutic measures.

## 9. Conclusions

Aging is associated with greater incidence of LUTS and ED in the male population. Pathophysiological mechanisms underlying concurrent development of the two conditions in the aging men remain largely elusive. We attempt to introduce the concept of pelvic arterial insufficiency as a unifying mechanism of LUTS-associated ED in the aging male. This concept is based on the notion that pelvic ischemia provokes simultaneous detrimental changes in the bladder, prostate, and penis. Our concept is supported by observations in animal models of pelvic atherosclerosis showing that pelvic ischemia engenders concurrent structural and functional modifications in the bladder, prostate, and penis. Molecular mechanisms activated by pelvic ischemia involve downstream pathways that elicit subcellular changes and lead to overactive smooth muscle contraction, impairment of smooth muscle relaxation, and activation of degenerative responses. These changes contribute to concomitant development of detrusor overactivity, excessive prostatic contractions, and penile erectile dysfunction. Further insight into the role of pelvic ischemia in LUTS-associated ED may change guideline recommendations and could lead to precise diagnostic markers, more effective therapeutic strategies, and a paradigm shift in the management of male patients with LUTS and ED.
